# Jane and Eliza Wigham

**Published:** 1889-01-05

**Authors:** 


### EDITOR'S LETTER-BOX.

[Our Correspondents are reminded that prolixity is a great bar to publication, and that brevity of style and conciseness of statement greatly facilitate early insertion.]

### JANE AND ELIZA WIGHAM.

I SEE that in THE HOSPITAL of the 22nd ult. thou dost speak with much praise of our dear sister Jane Wigham, who has recently gone to her rest; but as Jane has been an invalid for some time, I think thou must mean her daughter, Eliza Wigham, who is unceasingly active in good works. While we submit to the will of the Lord who has called the mother to Himself, we pray that it may be long before He takes Eliza Wigham from among us; not only because of her unwearying tenderness and charity for the unfortunate, the erring, and the sorrowful, but because it is good to see on earth one whose countenance is marked by that peace and purity which belong to the citizens of Heaven. C. G. F.

